# An empirical evaluation of the estimation of inbreeding depression from molecular markers under suboptimal conditions

**DOI:** 10.1111/eva.13568

**Published:** 2023-06-28

**Authors:** Noelia Pérez‐Pereira, Humberto Quesada, Armando Caballero

**Affiliations:** ^1^ Centro de Investigación Mariña Universidade de Vigo, Facultade de Bioloxía Vigo Spain

**Keywords:** fitness, inbreeding coefficient, quantitative traits, ROH, SNPs

## Abstract

Inbreeding depression (ID), the reduction in fitness due to inbreeding, is typically measured by the regression of the phenotypic values of individuals for a particular trait on their corresponding inbreeding coefficients (*F*). While genealogical records can provide these coefficients, they may be unavailable or incomplete, making molecular markers a useful alternative. The power to detect ID and its accuracy depend on the variation of *F* values of individuals, the sample sizes available, and the accuracy in the estimation of individual fitness traits and *F* values. In this study, we used *Drosophila melanogaster* to evaluate the effectiveness of molecular markers in estimating ID under suboptimal conditions. We generated two sets of 100 pairs of unrelated individuals from a large panmictic population and mated them for two generations to produce non‐inbred and unrelated individuals (*F* = 0) and inbred individuals (full‐sib progeny; *F* = 0.25). Using these expected genealogical *F* values, we calculated inbreeding depression for two fitness‐related traits, pupae productivity and competitive fitness. We then sequenced the males from 17 non‐inbred pairs and 17 inbred pairs to obtain their genomic inbreeding coefficients and estimate ID for the two traits. The scenario assumed was rather restrictive in terms of estimation of ID because: (1) the individuals belonged to the same generation of a large panmictic population, leading to low variation in individual *F* coefficients; (2) the sample sizes were small; and (3) the traits measured depended on both males and females while only males were sequenced. Despite the challenging conditions of our study, we found that molecular markers provided estimates of ID that were comparable to those obtained from simple pedigree estimations with larger sample sizes. The results therefore suggest that genomic measures of inbreeding are useful to provide estimates of inbreeding depression even under very challenging scenarios.

## 1 INTRODUCTION

Wild and domestic small populations are often affected by inbreeding (see, e.g., Hasselgren & Norén, 2019), which can lead to a decline in the average fitness‐related traits, known as inbreeding depression (Doekes et al., 2021; Hedrick & Garcia‐Dorado, 2016; Lynch & Walsh, 1998, Chap. 10). Inbreeding depression (ID) is of paramount relevance for the resilience of populations and the success of breeding programs (Frankham, 2003, 2005; Robertson, 1961; Wright et al., 2008), and occurs primarily due to the increased homozygosity of (partially) recessive deleterious mutations that are concealed in heterozygosis in a non‐inbred population. Inbreeding depression can be estimated by calculating the slope of the linear regression of the phenotypic values of individuals on their inbreeding coefficients (Lynch & Walsh, 1998). These coefficients can be obtained from pedigree data (Wright, 1969). However, pedigrees are not available for many organisms, particularly in wild populations (Pemberton, 2008), and molecular markers can be used instead. With the increasing availability of a large number of molecular markers, such as single nucleotide polymorphisms (SNPs), it is now possible to estimate the genomic inbreeding coefficient of individuals without requiring knowledge of their pedigree‐based relationships (Goudet et al., 2018; Howard et al., 2017; Wang, 2014; Yengo et al., 2017). Numerous studies have demonstrated the growing applicability of genomic inbreeding in both wild and domestic populations (e.g., Antonios et al., 2021; Foster et al., 2021; Gagnon et al., 2019; Luigi‐Sierra et al., 2022; Saura et al., 2015; Townsend & Jamieson, 2013). In addition, genomic inbreeding has the advantage of providing a more precise measure of the relationships between relatives compared to the pedigree coefficient, as the latter gives just expected values (Howard et al., 2017; Kardos et al., 2016; Wang, 2016).

There are several measures of the inbreeding coefficient of individuals from genomic data. These measures can be obtained either on a SNP‐by‐SNP basis, which depend on allele frequencies (e.g., Li & Horvitz, 1953; VanRaden, 2008; Yang et al., 2010), or from runs of homozygosity (ROH), that is, regions of the genome that are homozygous for all or the vast majority of nucleotide bases (Broman & Weber, 1999; McQuillan et al., 2008). Numerous simulation and empirical studies have compared the different measures of inbreeding based on markers, showing that some of them are very appropriate estimators of inbreeding depression (Caballero et al., 2021, 2022; Keller et al., 2011; Nietlisbach et al., 2019; Wang, 2014; Yengo et al., 2017).

Both empirical and simulation studies indicate that estimates using data from different and relatively distant generations, implying a substantial variation in *F* values, increase the power to estimate ID (e.g., Bérénos et al., 2016; Doekes et al., 2019; Martikainen et al., 2017; Saura et al., 2015; Silió et al., 2013; Sumreddee et al., 2019). However, in many cases, historical data may not be available, and the *F* estimate is obtained using individuals from the contemporaneous population. In such situations, the variation in *F* values among individuals can be low, particularly for large sized populations, which may compromise the estimation of ID. Furthermore, the power to detect ID strongly depends on the sample sizes (Wang, 2014) and the reliability of the estimates of individual fitness, which can be subject to substantial error and sources of environmental variation due to their low heritabilities (see, e.g., Caballero, 2020, p. 57).

In this study, we aimed to evaluate inbreeding depression for two fitness traits of *Drosophila melanogaster* under restricted experimental conditions using individual *F* values estimated from molecular markers. Firstly, individuals were sampled from a large panmictic population at a single generation, which would usually lead to low variation in *F* values. Secondly, the sample size for analysis was rather limited with only 17 pairs of individuals. Lastly, the traits measured depended on both males and females, but only males were sequenced and had their inbreeding coefficients estimated. Our results show the usefulness of genomic measures of inbreeding for estimating inbreeding depression, despite the strong experimental limitations.

## 2 METHODOLOGY

In brief, we estimated the inbreeding depression rate for two fitness traits (pupae productivity and competitive fitness) in a large panmictic laboratory population of *D. melanogaster*, following a full‐sib mating design. One hundred non‐inbred pairs of individuals and 100 inbred pairs (full‐sib progeny) were established and scored for the traits when possible. This allowed an estimate of the inbreeding depression to be obtained from the expected pedigree estimates of inbreeding (0 and 0.25 respectively). DNA was individually extracted for a set of 17 males from the non‐inbred pairs, and 17 males from the inbred pairs, and whole‐genome sequencing was carried out obtaining a large number of SNPs. Estimates of the inbreeding depression rate were then obtained from the linear regression of the individual fitness values on the genomic measures of inbreeding obtained by means of two estimators. The estimated ID rates obtained from the markers were compared with those obtained from the simple pedigree values of expected inbreeding considering a much larger sample size.

### 2.1 Base population, breeding design and fitness traits evaluated

**FIGURE 1:**
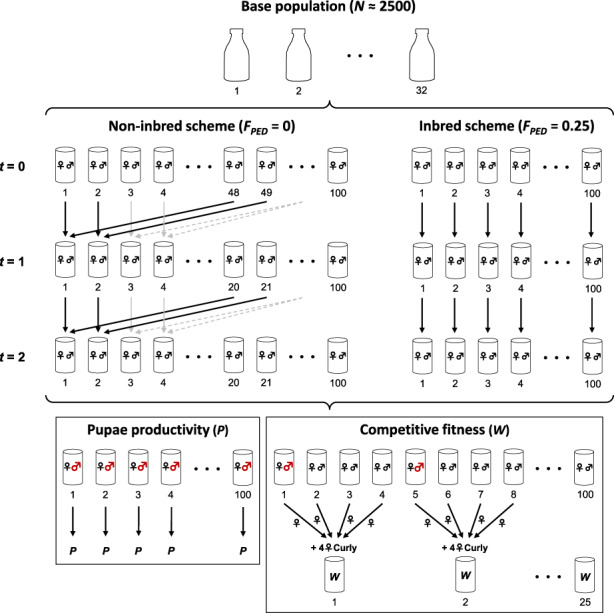
Experimental design followed to evaluate fitness and inbreeding depression. Two schemes were derived from the base population to achieve an expected inbreeding coefficient of *F*_PED_ = 0 and *F*_PED_ = 0.25. Fitness was measured as mean pupae productivity (*P* for both the non‐inbred and inbred schemes) produced after 11 days since the last mating (*t* = 2), and as competitive fitness (*W* for both the non‐inbred and inbred schemes, as the ratio of Wild‐type to Curly‐type offspring) 14 days after placing those mated females at *t* = 2 in the presence of curly females in groups of four (i.e., four Wild‐type females plus four Curly‐type females). Sequenced males are shown in red (see main text for details on how males were selected for sequencing).

A population of *D. melanogaster* was founded in October 2018, derived from a wild population located in a wine cellar in Ponteareas (Pontevedra, Spain). This population was maintained in the laboratory for 50 generations at a constant population size of around *N* ≈ 2500 individuals, distributed in 32 bottles with ~80 individuals per bottle. The laboratory conditions were kept invariant over generations, with a constant temperature of 25°C and continuous lighting. To ensure a near panmictic scenario, circular mixing of individuals from consecutive bottles was carried out each generation. Individuals introduced in each bottle were removed after a week in order to avoid the overlap of generations. A summary of the overall experimental design is presented in Figure 1.

An evaluation of fitness and inbreeding depression was carried out at generation 50, by sampling at least three virgin females and three males from each bottle and randomly placing pairs of individuals in single vials, obtaining a total of 100 breeding pairs with the subsequent separate offspring. This was considered a transition generation to exclude maternal effects (generation *t* = 0). Two separate schemes were derived from the offspring: First, a non‐inbred scheme, where individuals were mated randomly in individual vials for two generations (100 breeding pairs) but avoiding inbred matings. This was achieved by mating individuals from distant vials. For example, at generation *t* = 1, a female from vial 1 was mated with a male from vial 48, a female from vial 2 with a male from vial 49, and so on, following a circular scheme (see Figure 1). Analogously, at generation *t* = 2, a female from vial 1 was mated with a male from vial 20, a female from vial 2 with a male from vial 21, and so on. Thus, adult individuals at generation *t* = 2 were expected to be unrelated with an expected inbreeding coefficient *F*_PED_ = 0, relative to the base population, in this case the generation 50 of the large population from which the experiment started. Second, an inbred scheme was derived by mating full‐sib individuals for two generations. Thus, adult individuals had an expected inbreeding coefficient *F*_PED_ = 0.25 at generation *t* = 2, relative to the base population, but individuals from different families were also expected to be unrelated among each other.

Two fitness‐related traits were simultaneously evaluated at generation *t* = 2 for the two sets of individuals analysed. On the one hand, pupae productivity (*P*) was obtained as the mean number of pupae produced from each breeding pair in each scheme after 11 days from mating (*t* = 2). On the other hand, to evaluate competitive fitness (hereafter referred simply to as fitness, *W*), females were taken from the vials of the two schemes 3 days after the mating (*t* = 2) and were placed together with Curly‐type females (previously randomly mated in bottles) in groups of eight females per vial, that is, four independent wild‐type females (i.e., from four different vials) plus four Curly‐type females per vial (see Figure 1). In the inbred scheme (*F*_PED_ = 0.25), it was not possible to include fully unrelated wild‐type females in all evaluation vials, and about half of the vials included two wild‐type females which were cousins, and therefore had an expected coancestry coefficient of *f* = 0.25. After 14 days, wild‐type offspring and Curly‐type offspring were counted from each evaluation vial and fitness (*W*) was measured as the ratio Wild/(Curly + 1). Note that both fitness measures are complex traits involving mating success, parental fecundity and egg‐to‐pupae viability in the case of productivity (*P*), and also egg‐to‐adult viability including competition against the Curly strain in the case of fitness (*W*; see, e.g., López‐Cortegano et al., 2016). Thus, both traits measured in the progeny depend on both male and female parents. In the case of pupae productivity, it was previously shown that about half the ID shown in the trait is due to the female parents (Avila et al., 2013).

The male parents from about 60 randomly chosen non‐inbred pairs and about 60 inbred pairs at generation *t* = 2 were taken to be frozen for later DNA extraction and sequencing (see next section), but only a subset of 17 non‐inbred and 17 inbred males were sequenced. Female parents were not considered for sequencing to avoid offspring contamination. Note that the individual pair productivity (*P*) corresponds to the pair, that is, to the male (sequenced) and the female (not sequenced) of each particular vial. In the case of competitive fitness against Curly flies (*W*), each individual value encompasses the fitness of the female progeny derived from four pairs, from which only one male was sequenced and three males and four females were not sequenced. The complete set of data including the fitness trait values from the evaluated flies indicating the males sequenced along with additional information, are shown in Supplemental File S1.

The inbreeding depression rate (*ID*_PED_) for *P* and *W* was obtained from the slope of the linear regression of the natural logarithm of the individual phenotypic values, log(*P*) and log(*W*), on *F*_PED_. Estimates were obtained for each trait including all pairs of individuals for which phenotypic values were available (*ID*_PED_).

### 2.2 DNA extraction, sequencing and SNP calling

As mentioned above, breeding males from generation *t* = 2 were removed from their corresponding vials 3 days after mating, frozen with liquid nitrogen and individually stored at −80°C until DNA extraction. DNA extraction was carried out using the Gentra Puregene Cell Kit (Qiagen) with some modifications, including an RNase step. From all males frozen, we randomly chose 17 from the non‐inbred scheme (*F*_PED_ = 0) and 17 from the inbred scheme (*F*_PED_ = 0.25) from those meeting the following requirements: (i) a minimum DNA concentration of 10 ng/μL (in a final volume of 10 μL), measured in a Qubit fluorimeter (Thermo Fisher Scientific), (ii) a minimum DNA integrity number (DIN) of 8 measured in an Agilent TapeStation system, and (iii) the presence of a phenotypic value for both pupae productivity (*P*; those individuals showing a phenotypic value equal to zero were excluded) and fitness (*W*; avoiding males from the same group, that is, only one male was selected per group of four families as indicated in Figure 1, where the males that were sequenced are represented in red). Nextera XT DNA library preparation and genome sequencing were carried out at the NimGenetics Genomics Service, preparing a single Illumina library for each individual fly. A total of 34 DNA samples (17 from individuals with *F*_PED_ = 0 and 17 from individuals with *F*_PED_ = 0.25) were used for 2 × 150 bp paired‐end sequencing on the Illumina Novaseq 6000 instrument to a mean read depth of ~50 times.

Paired‐end reads were processed from the FASTQ files to finally obtain the Variant Call Format (VCF) files with data for filtered biallelic autosomic SNPs. Briefly, adapters were removed with Trimmomatic (Bolger et al., 2014) using the Nextera adapter list, and reads were filtered according to their quality and size with the ERNE‐FILTER tool (default options except for ‐‐*min‐size* = 36; Del Fabbro et al., 2013). The resulting reads were mapped to the v. 6.14 *D. melanogaster* reference genome from Flybase (www.flybase.org/) using the BWA‐MEM algorithm from the Burrows‐Wheeler Alignment (BWA) software (Li, 2013). After mapping, PCR duplicates were removed and the alignments filtered for a minimum map quality score of 20 with SAMtools (Danecek et al., 2021). Potential variant sites were called with the HaplotypeCaller tool from the Genome Analysis Toolkit (GATK; Van der Auwera & O'Connor, 2020). The resulting GVCF (Genomic Variant Call Format) files were combined into one using the GATK CombineGVCFs tool. The GATK GenotypeGVCFs tool was used to obtain genotype information for all variants. Multiallelic and monomorphic SNPs, as well as indels and those SNPs within 10 bp of an indel, were removed with BCFtools (Danecek et al., 2021). Highly repeated sites and low information areas were also excluded with VCFtools (Danecek et al., 2011). Finally, SNPs were (hard) filtered with the GATK VariantFiltration tool applying the recommended presets if: QD <2.0, FS >60, SOR >3.0, MQRankSum <−12.5, ReadPosRankSum <−8.0 and QUAL <30.0. From the VCF file with information on the SNPs that passed the filtering, PED and MAP files were obtained with PLINK (version 1.9; Purcell et al., 2007).

Around 1,140,000 SNPs from autosomal chromosomes were finally available for inbreeding analysis. Detailed information about the sequencing quality parameters, such as the mean mapping quality (58.5 on average), mean coverage (49× on average), percentage of the genome covered by at least one read (93.5% on average) and total number of mapped bases (5.4 × 10^9^ on average) for each individual can be found in Supplemental File S1, along with DNA extraction data.

### 2.3 Estimation of inbreeding depression from molecular inbreeding measures

Two estimators of the inbreeding coefficient were obtained with PLINK (version 1.9) from the SNPs available for individuals with *F*_PED_ = 0 and for individuals with *F*_PED_ = 0.25 separately. The first estimator is a SNP‐by‐SNP estimator obtained with the command *‐ibc* (equivalent to the *‐ibc* from GCTA; Yang et al., 2011) and calculated as

$$F_{\mathrm{YAN}}=\frac{1}{S}\sum_{k=1}^{S}\frac{x_{k}^{2}-\left(1+2p_{k}\right)x_{k}+2p_{k}^{2}}{2p_{k}\left(1-p_{k}\right)},$$

where *S* is the total number of markers, *x*_*k*_ the number of minor alleles of marker *k* (0, 1 or 2 copies) and *p*_*k*_ the current frequency of the minor allele of marker *k* (Yang et al., 2010). *F*_YAN_ (called *F* ^III^ in PLINK) is based on the correlation between uniting gametes.

Estimates of *F* were also obtained from runs of homozygosity (ROH), calculated as

$$F_{\mathrm{ROH}}=\frac{\sum L_{\mathrm{ROH}}}{L_{\text{auto}}},$$

where ∑*L*_ROH_ is the sum of the lengths of all ROHs (above a specified minimum length) that cover the genome of an individual, and *L*_auto_ the length of the autosomal genome covered by SNPs (McQuillan et al., 2008), that is, 119 Mb for autosomal chromosomes considering that around 90% of the reference genome was sequenced. First, LD‐based pruning was carried out to remove highly linked SNPs (*r* ^*2*^ > 0.9) using the *‐‐indep‐pairwise 50 5 0.9* PLINK option, as recommended by Howrigan et al. (2011). Around 420,000 SNPs remained after pruning. Then, *F*_ROH_ was obtained with the *‐‐homozyg* command using the default options to define a ROH, among which stands out a minimum number of SNPs of 100, a minimum density of 1 SNP per 50 Kb and a scanning window of 50 SNPs (with 1 heterozygote allowed per window). Two minimum ROH lengths were applied, 0.1 Mb (*F*_ROH‐0.1_) and 1 Mb (*F*_ROH‐1_).

Among the various available measures for estimating inbreeding, these two molecular measures have been shown to be particularly reliable for estimating the rate of ID (Caballero et al., 2021, 2022; Keller et al., 2011; Nietlisbach et al., 2019; Wang, 2014; Yengo et al., 2017). The inbreeding depression rate (*ID*_YAN_, *ID*_ROH‐0.1_ and *ID*_ROH‐1_) was obtained from the slope of the linear regression of the natural logarithm of the phenotypic values for the two traits evaluated (pupae productivity, log(*P*), and fitness, log(*W*)) on each of the estimated inbreeding coefficients. The statistical significance of the ID estimates was obtained with three methods. First, from the parametric error attached to the regression coefficients obtained. Second, using a bootstrap sampling over individuals (10,000 samples of the same size as the original data). And third, with a randomization test in R. For this latter, for each ID estimate to be tested, phenotypic values were first randomized among individuals using the function *sample()*, and an estimate of ID was then obtained from the linear regression of the randomized phenotypic values on *F* using the function *lm()*. These two steps were repeated 10,000 times, and *p*‐value*s* were calculated as (*N*_IDr>IDobs_ + 1)/10,001, where *N*_IDr>IDobs_ is the number of ID values resulting from the randomization test that resulted in greater inbreeding depression than the observed ID. Pearson correlations (and *p*‐value*s*) between *F* estimates and the phenotypic values, as well as between *F* estimates with each other, were obtained with the function *cor.test()* in R.

### 2.4 Expectation of the rate of inbreeding depression estimated by the molecular inbreeding measures given the experimental design

Because *F* estimates from molecular markers were only obtained for males, while the phenotypic values correspond to the pair, that is, the male and the female, in the productivity measure, and to four pairs in the case of fitness, we might expect a partial estimate of the pedigree ID. Following the results from Avila et al. (2013), we assume that both traits depend equally on both parents. In that case, estimates from unrelated individuals (*F*_PED_ = 0) are expected to be one half the value estimated if both sexes were considered in the case of productivity, and one eight in the case of fitness. Analogously, estimates from full‐sib individuals (*F*_PED_ = 0.25) are expected to be a fraction around 7/8 and 9/32 for productivity and fitness, respectively (see Supplemental File S2).

## 3 RESULTS

Mean molecular measures of the inbreeding coefficient are shown in Table 1. SNP‐by‐SNP estimates (*F*_YAN_) provided values close to zero and slightly negative for individuals with *F*_PED_ = 0, as expected. ROH‐based estimates provided larger values, especially when a minimum length of 0.1 Mb (*F*_ROH‐0.1_) was applied, compared to a minimum length of 1 Mb (*F*_ROH‐1_) and, to a larger extent for individuals with *F*_PED_ = 0.25, as expected. Standard errors (SEs) were also larger for shorter ROH lengths. For all estimates, standard deviations between *F* values of individuals were larger for *F*_PED_ = 0.25 than for *F*_PED_ = 0. Table 2 gives the information of the ROH fragments found. Individuals with *F*_PED_ = 0.25 presented a larger proportion of the genome with ROH, with 5% more fragments per individual on average than individuals with *F*_PED_ = 0 for a minimum ROH length of 0.1 Mb, and about twice the number of fragments when a minimum ROH length of 1 Mb was applied. No differences in SNP density within ROH were observed between ROH lengths and origin of individuals.

**TABLE 1:** Mean inbreeding coefficient (*F* ± SE) obtained from different molecular measures (see text) and for the two sets of individuals (non‐inbred, *F*_PED_ = 0; and inbred, *F*_PED_ = 0.25).

	*F*_YAN_	*F*_ROH‐0.1_	*F*_ROH‐1_
*F*_PED_ = 0	−0.0520 ± 0.0154 (SD: 0.064)	0.1477 ± 0.0181 (SD: 0.075)	0.0518 ± 0.0119 (SD: 0.049)
*F*_PED_ = 0.25	0.0346 ± 0.0245 (SD: 0.101)	0.2143 ± 0.0256 (SD: 0.105)	0.1181 ± 0.0199 (SD: 0.082)

*Note*: Standard deviations (SD) of *F* values among individuals are shown in parentheses.

**TABLE 2:** Mean number of ROH identified per individual, mean length in kilobases, mean number of SNPs in a ROH and mean SNP density (number of SNPs per kb within ROH), for the two sets of individuals (non‐inbred, *F*_PED_ = 0; and inbred, *F*_PED_ = 0.25; ±SE).

	ROH/ind.	Length (kb)	SNPs	SNP density
*F*_PED_ = 0				
*F*_ROH‐0.1_	36.59 ± 3.23	480.45 ± 20.14	1781.73 ± 77.18	3.98 ± 0.08
*F*_ROH‐1_	3.65 ± 0.87	1690.29 ± 82.38	5385.76 ± 363.70	3.48 ± 0.25
*F*_PED_ = 0.25				
*F*_ROH‐0.1_	38.47 ± 2.82	662.74 ± 27.77	2534.95 ± 134.34	3.92 ± 0.09
*F*_ROH‐1_	7.88 ± 1.21	1782.28 ± 70.42	6685.19 ± 466.50	3.82 ± 0.19

Abbreviations: ROH, runs of homozygosity; SNPs, single nucleotide polymorphisms.

Pearson correlations between *F* estimates (Table 3; *p*‐values in Table S1, Supplemental File S3) were very high in all cases (>0.70), but to a greater extent for individuals with *F*_PED_ = 0.25. Table 4 presents the Pearson correlations between molecular *F* values and the two fitness traits, Log(*P*) and Log(*W*). Overall, *F*_YAN_ showed the highest (negative) correlation with Log(*P*) and Log(*W*). Molecular *F* estimates were more correlated with Log(*P*) than with Log(*W*), at least for individuals with *F*_PED_ = 0, as expected given the experimental design, although the opposite occurred with individuals with *F*_PED_ = 0.25. Despite these trends, correlations were not significant in any case (*p*‐value >0.08; Table S2 in Supplemental File S3).

**TABLE 3:** Pearson correlation between different molecular estimates of the inbreeding coefficient (*F*; see text) and for the two sets of individuals (non‐inbred, *F*_PED_ = 0 over the diagonal; and inbred, *F*_PED_ = 0.25, below the diagonal).

	*F*_YAN_	*F*_ROH‐0.1_	*F*_ROH‐1_
*F*_YAN_	–	0.867	0.775
*F*_ROH‐0.1_	0.879	–	0.905
*F*_ROH‐1_	0.880	0.968	–

*Note*: *p*‐values < 0.001 for all estimates.

**TABLE 4:** Pearson correlation between different molecular measures of the inbreeding coefficient (*F*; see text) and the phenotypic values for two fitness traits: pupae productivity (*P*) and fitness (*W*), for the two sets of individuals (non‐inbred, *F*_PED_ = 0; and inbred, *F*_PED_ = 0.25).

	*F*_YAN_	*F*_ROH‐0.1_	*F*_ROH‐1_
*F*_PED_ = 0			
Log(*P*)	−0.3989	−0.2163	−0.1762
Log(*W*)	−0.1539	−0.0329	−0.1129
*F*_PED_ = 0.25			
Log(*P*)	−0.3278	−0.3448	−0.2975
Log(*W*)	−0.4339	−0.4241	−0.3937

*Note*: *p*‐values > 0.082.

**FIGURE 2:**
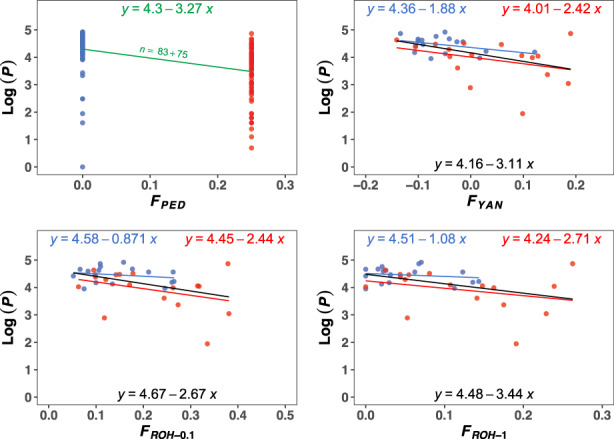
Linear regression of the natural logarithm of the phenotypic values for pupae productivity (Log(*P*)) on different estimates of the inbreeding coefficient. The upper left graph shows the regression of the phenotypic values of all individuals experimentally evaluated on pedigree‐based estimates (i.e., *F*_PED_ = 0 and *F*_PED_ = 0.25). The number of samples is indicated by *n*, being the sum of individuals with *F*_PED_ = 0 and *F*_PED_ = 0.25. The other graphs show the regression of the phenotypic values (individuals that were sequenced) on the molecular estimates of the inbreeding coefficient (*F*_YAN_, *F*_ROH‐0.1_ and *F*_ROH‐1_). Colors indicate the origin of the individuals for which *F* was estimated, that is, individuals with *F*_PED_ = 0 (blue) or *F*_PED_ = 0.25 (red). Regressions considering both set of data are shown in black. Standard errors of regression coefficients are shown in Table 5.

Inbreeding depression (ID) was obtained as the slope of the linear regression of the phenotypic values, Log(*P*) and Log(*W*), on the inbreeding coefficient estimates. All estimates of the rate of inbreeding depression are summarized in Table 5. Regarding Log(*P*) (Figure 2), pedigrees provided an inbreeding depression rate of *ID*_PED_ = −3.27 (*p*‐value = 10^−4^; upper left graph). The average productivity in the non‐inbred population was 85.66 ± 3.39 pupae, while that in the inbred population was 44.68 ± 3.32 pupae, almost half the former.

**TABLE 5:** Observed mean inbreeding depression rates (*ID* ± SE) using the different molecular measures of inbreeding and their expected values (*E*[*ID*_MOL_]) as fractions of the pedigree ID (*ID*_PED_) for the two traits (productivity *P*, and fitness *W*) and for the two sets of individuals (non‐inbred, *F*_PED_ = 0; inbred, *F*_PED_ = 0.25; and with both sets of data).

Fitness trait	*F*_PED_	*ID*_PED_	Fraction	*E*[*ID*_MOL_]	*ID*_YAN_	*ID*_ROH‐0.1_	*ID*_ROH‐1_
log(*P*)	0	−3.27^a^ ± 0.54	1/2	−1.64	−1.88^a^ ± 1.11	−0.87 ± 1.01	−1.08 ± 1.55
	0.25		7/8	−2.86	−2.42 ± 1.80	−2.44 ± 1.72	−2.71 ± 2.24
	Both				−3.11^a^ ± 1.03	−2.67^a^ ± 1.04	−3.44^a^ ± 1.34
log(*W*)	0	−3.75^a^ ± 1.44	1/8	−0.47	−1.95 ± 3.23	−0.36 ± 2.78	−1.85 ± 4.20
	0.25		9/32	−1.05	−5.50^a^ ± 2.95	−5.16^a^ ± 2.84	−6.15 ± 3.71
	Both				−5.57^a^ ± 1.89	−4.63^a^ ± 1.92	−6.50^a^ ± 2.43

^a^ Values significantly different from zero.

Marker‐based IDs are presented in the remaining graphs of Figure 2, with color indicating the origin of individuals, that is, *F*_PED_ = 0 (blue) or *F*_PED_ = 0.25 (red). Despite the small number of individuals evaluated (only 17 per scheme) and the consequent noise, an inbreeding depression trend was observed in all cases, even when the individuals had an expected inbreeding coefficient *F*_PED_ = 0. As indicated above, because the estimated inbreeding coefficient referred to just one individual of each pair, the expected ID from markers would be half the value from pedigrees in the case of *F*_PED_ = 0 (i.e., *ID*_PED_/2 = −1.64; Table 5). In fact, the molecular estimate of ID from *F*_YAN_ was rather close to this figure (*ID*_YAN_ = −1.88), whereas ROH measures provided lower estimates (*ID*_ROH‐0.1_ = −0.87 and *ID*_ROH‐1_ = −1.08). In the case of individuals with *F*_PED_ = 0.25, marker‐based IDs were larger than those obtained from individuals with *F*_PED_ = 0, as expected because the estimation of ID would be ⅞ × *ID*_PED_ = −2.86 (Supplemental File S2). The estimates from molecular markers were rather close to this expectation (*ID*_YAN_ = −2.42; *ID*_ROH‐0.1_ = −2.44; *ID*_ROH‐1_ = −2.71). Because of the considerable noise in the samples, none of the estimates of ID were significantly different from zero, except that for *ID*_YAN_, which reached significance with the bootstrap method for individuals with *F*_PED_ = 0 (*p*‐value = 0.03). All *p*‐value*s* are shown in Supplemental File S3 (Table S3). Although there were some differences between methods, there was a general concordance between them.

**FIGURE 3:**
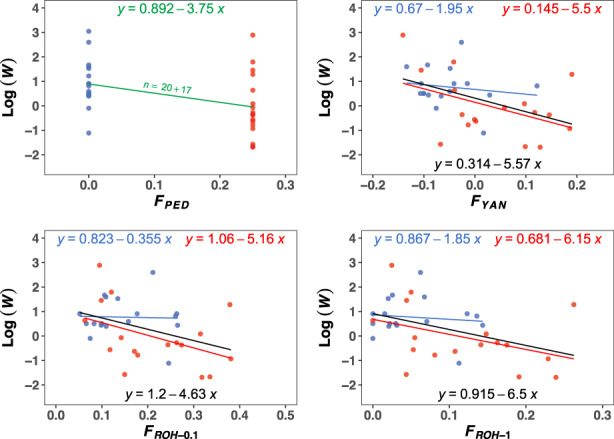
Linear regression of the natural logarithm of the phenotypic values for fitness (Log(*W*)) on different estimates of the inbreeding coefficient. The upper left graph shows the regression of the phenotypic values of all individuals experimentally evaluated on pedigree‐based estimates (i.e., *F*_PED_ = 0 and *F*_PED_ = 0.25). The number of samples is indicated by *n*, being the sum of individuals with *F*_PED_ = 0 and *F*_PED_ = 0.25. The other graphs show the regression of the phenotypic values (individuals that were sequenced) on the molecular estimates of the inbreeding coefficient (*F*_YAN_, *F*_ROH‐0.1_ and *F*_ROH‐1_). Colors indicate the origin of the individuals for which *F* was estimated, that is, individuals with *F*_PED_ = 0 or *F*_PED_ = 0.25. Regressions considering both set of data are shown in black. Standard errors of regression coefficients are shown in Table 5.

Figure 3 shows analogous results for Log(*W*). Pedigrees provided an inbreeding depression rate of *ID*_PED_ = −3.75 (*p*‐value = 0.01; upper left graph). For this trait, the estimates obtained from molecular markers are expected to be around ⅛ of the actual ID of the population, (i.e., *ID*_PED_/8 = −3.75/8 = −0.47; Table 5) when estimated from individuals with *F*_PED_ = 0. Estimates were very variable across estimators with values larger (*ID*_YAN_ = −1.95; *ID*_ROH‐1_ = −1.85) and lower (*ID*_ROH‐0.1_ = −0.36) than the expectations. In the case of individuals with *F*_PED_ = 0.25 the expectation would be around 9/32 × *ID*_PED_ = −1.05 (Supplemental File S2; Table 5) and all estimates from molecular markers were substantially larger than that value (*ID*_YAN_ = −5.5; *ID*_ROH‐0.1_ = −5.16; *ID*_ROH‐1_ = −6.15). However, there was a large noise in the samples and estimates were only significantly different from zero for *ID*_YAN_ and *ID*_ROH‐0.1_ for individuals with *F*_PED_ = 0.25 (*p*‐value = 0.04).

When the estimates of ID were obtained grouping the two sets of data (i.e., with 34 samples instead of 17), these were in general similar to those obtained for individuals with *F*_PED_ = 0.25 (black regression lines in Figures 2 and 3), with the difference that now all ID estimates were significantly different from 0 irrespective of the test method (*p*‐value <0.041; Table S3 in Supplemental File S3), and were closer to the ID estimates obtained from *F*_PED_ measures in the case of productivity (Table 5).

The above results were obtained considering whole genome sequencing data. In order to assess the performance of the estimators when a lower number of SNPs is available, mean *F* values and ID estimates were obtained from a random subset of SNPs (from 500,000 down to 5000; Tables S4 and S5 in Supplemental File S3). Mean *F* values were not particularly affected by the number of SNPs, except for ROH‐based estimates (Table S4). *F*_ROH‐1_ values were increased for a decreasing number of SNPs down to 20,000, while *F*_ROH‐0.1_ were slightly decreased. For a number of SNPs below 20,000, both estimates offered virtually the same values, as short ROH were more difficult to detect (e.g., for *F*_ROH‐0.1_ with 5000 SNPs, the minimum length of a segment detected was around 2 Mb; not shown). Analogously, ID estimates were not particularly affected by the number of SNPs when using SNP‐by‐SNP estimators (Table S5), although an increase in the SE was observed when the number of SNPs was reduced. ROH‐based estimates provided lower values of ID as the number of SNPs decreased down to 20,000 (to a larger extent with *F*_ROH‐1_), but estimates of ID were similar for different sets of SNPs, except for ROH‐based estimates for individuals with *F*_PED_ = 0 for Log(*W*), which could be explained by the low correlation expected between the male and the corresponding phenotypic value. In fact, ID measures for Log(*W*) had the largest SEs. With a number of SNPs of 5000–10,000, ROH‐based estimates tended to provide increased estimates of ID when obtained from individuals with *F*_PED_ = 0, or decreased estimates when obtained from individuals with *F*_PED_ = 0.25.

Finally, to assess the relevance of those SNPs segregating at low frequencies, a minimum allele frequency (MAF) of 0.05 was applied in PLINK to estimate *F* and ID (Tables S4 and S5 in Supplemental File S3). ID values were barely affected, with *ID*_YAN_ showing a slight reduction. This is expected since with only 17 sampled individuals, only SNPs present in single copy are expected to be lost after MAF pruning. However, for log(*W*) values from individuals with *F*_PED_ = 0, we observed a reduction in *ID*_ROH‐0.1_ from −0.355 to −0.034. We also did not find large differences in ROH‐based IDs when modifying the default parameters in PLINK (Table S6 in Supplemental File S3).

## 4 DISCUSSION

The power of molecular markers to estimate the rate of inbreeding depression is influenced by several factors, such as the level of variation in individual *F* values, the accuracy of *F* estimation, the sample sizes available, and the reliability of fitness measures. Empirical studies on the performance of molecular markers in estimating ID typically involve samples from multiple generations, which allows for a wide range of inbreeding coefficients among individuals, which facilitates estimation accuracy. For example, most studies include pedigrees spanning seven to 12 pedigree generations (Antonios et al., 2021; Bérénos et al., 2016; Doekes et al., 2019; Ferenčaković et al., 2017; Makanjuola et al., 2020; Martikainen et al., 2017; Zhang et al., 2022), with some studies covering more than 20 generations (Saura et al., 2015; Silió et al., 2013; Sumreddee et al., 2019) and a few spanning fewer than five generations (Hidalgo et al., 2021; Pryce et al., 2014). These studies are also characterized by involving large sample sizes, exceeding 1000 individuals and sometimes reaching over 20,000 individuals (Doekes et al., 2019; Makanjuola et al., 2020). While results vary due to species differences, trait variation, and study‐specific factors, molecular estimators generally outperform pedigree‐based ones. When both measures are statistically significant, pedigree and molecular estimates of ID tend to be close to each other (e.g., Doekes et al., 2019; Makanjuola et al., 2020; Silió et al., 2013; Sumreddee et al., 2019), but results may vary otherwise, which is often the case.

In many situations, particularly in the case of large natural populations, it may not be possible to obtain samples from multiple generations, and these belong to a single one, generally allowing for little variation in inbreeding coefficients across individuals, which may be only obtainable for a limited number of sampled individuals. Moreover, obtaining precise estimates of individual fitnesses can be challenging. Here, we carried out an experimental design with *D. melanogaster* under suboptimal conditions to estimate ID using molecular markers. We considered a small sample of individuals obtained from a single generation of a large panmictic population, with the assumption that the individuals are mostly unrelated and with a low variation in their inbreeding coefficients. Moreover, ID estimates were obtained for traits which depend on both parents (Avila et al., 2013), while inbreeding measures were taken only from the male parent. In addition, in the case of fitness, the phenotypic values were the mean of four different families, of which only one male parent was sequenced. It also should be noted that *D. melanogaster* has a characteristic small genome (180 Mb) with lack of recombination in males and a total genome length of about 1.5 Morgans. Despite these limitations, our results indicate that it is possible to estimate inbreeding depression using molecular markers even in such extreme scenarios. Although many of the estimates were nonsignificant with the small sample sizes considered, they all showed a trend compatible with inbreeding depression and estimated values of ID close to those expected, at least for productivity. All estimates became significant when we pooled the two sets of individuals (inbred and non‐inbred) and obtained a higher variation in individual *F* values. Thus, our study highlights the potential of molecular markers for estimating inbreeding depression in situations with low sample sizes of unrelated individuals obtained from a single generation.

We focused on using molecular markers to estimate inbreeding depression in *D. melanogaster*, a commonly used model species. We found rates of inbreeding depression of 3.27 for productivity and 3.75 for competitive fitness. The estimate for productivity was obtained from the regression of log productivities on *F* values in order to compare pedigree and molecular estimates of ID. An alternative calculation, carried out in other studies, is to obtain the estimate from the ratio of the log mean productivities. In this case, ID = log(*P*_*F*PED=0_/*P*_*F*PED=0.25_)/0.25 = log(85.66/44.68)/0.25 = 2.60, which is lower than the former value (3.27). The estimate would be still a bit lower (2.08) if the assumed expected inbreeding coefficient attached to the trait is 0.3125 rather than 0.25, if we assume that productivity depends equally on the parents inbreeding (*F* = 0.25) and that of their progeny (*F* = 0.375). This implies that the rate of ID for productivity is a decline of about 2% of the mean for each 0.01 increase in inbreeding. This value is higher than previous equivalent estimates obtained for the same trait in other laboratory populations: 0.85 for a population maintained in the lab for 112 generations, and 1.40 for a different population maintained in the lab for 50 generations (Pérez‐Pereira et al., 2021). This suggests that the population analysed in the present study (also maintained in the lab for 50 generations and with analogous census size as the other populations) had a larger amount of deleterious variation at the time of analysis than the other ones. The estimates of the rate of ID found in the present experiment (around 2–4) are comparable to those obtained in mammals. For example, estimates of the inbreeding load (which equals the rate of ID in the absence of selection) for fecundity, first year survival and survival to sexual maturity in wild mammals and birds, compiled by O'Grady et al. (2006), are 1.97, 1.18 and 2.98 lethal equivalents, respectively, and those compiled by Nietlisbach et al. (2019) for survival to sexual maturity in wild vertebrate populations are on average of 3.5 lethal equivalents. Likewise, the estimated inbreeding load in Soay sheep annual survival based on genomic data is 2.285 lethal equivalents (Stoffel et al., 2021). Some estimates with much larger values are cited by Hedrick and Garcia‐Dorado (2016). Average estimates of ID in Livestock for reproduction/survival traits are, however, much lower (0.22; Leroy, 2014).

The rate of inbreeding depression for fitness traits is influenced by several factors, including the haploid rate of deleterious mutations (*U*), the selection coefficient against mutant homozygotes (*s*) and their dominance coefficients (*h*). These parameters can be estimated using mutation‐accumulation (MA) experiments, which involve propagating populations under conditions that allow mutations to accumulate randomly over generations. Halligan and Keightley (2009) compiled estimates for these parameters from various MA studies for several species, including *D. melanogaster*. The median *U* estimate for Drosophila (based on 16 estimates, mostly for viability) is 0.061, which is slightly higher than the corresponding value for Arabidopsis (0.053; based on six estimates) and much higher than that for nematodes (0.004; based on nine estimates). The median *s* values for Drosophila, Arabidopsis and nematodes are 0.11, 0.20 and 0.22, respectively. These results suggest that Drosophila has a somewhat higher mutation rate with lower mutational effects compared to other species, although there is considerable variation across estimates. Estimating the dominance coefficient of deleterious mutations is a challenging task, and available estimates are both limited and variable. However, mean estimates for Drosophila, yeast, *Caehorhabditis elegans*, and *Arabidopsis thaliana* are around 0.2–0.3 (Agrawal & Whitlock, 2011; Caballero, 2006, 2020, p. 158; Manna et al., 2011), with lower values for strongly deleterious mutations than for milder ones (Agrawal & Whitlock, 2011; Caballero, 2006; Huber et al., 2018; Mukai et al., 1972; Simmons & Crow, 1977). It should be noted that the above estimates refer only to a fraction of mutations of relatively large effect, which can be detected in MA experiments (García‐Dorado et al., 2004; Halligan & Keightley, 2009). This class of mutations, along with that of lethals, which arise at a rate of about 0.015 (Simmons & Crow, 1977), is expected to be the most relevant when it comes to assessing the impact of inbreeding depression and the evolution of fitness in small populations and short periods of time, as shown by comparisons between computer simulations and empirical results (e.g., Caballero et al., 2002; Caballero & Keightley, 1998; Pérez‐Pereira et al., 2021). In Drosophila, about half the inbreeding load observed is due to lethal mutations (Lynch & Walsh, 1998, p. 281; Simmons & Crow, 1977). Estimates of the rate of deleterious mutations (*U*) obtained from molecular data, such as the comparison of nucleotide divergences between closely related species, typically include a much broader spectrum of mutation effects. For example, Haag‐Liautard et al. (2007) estimated *U* to be around 0.6 in Drosophila and Keightley (2012) obtained an estimate of 1.1 in humans. Similarly, studies of the frequency spectrum of human populations suggest that the mean effect of homozygous mutations is about 0.013, with a very leptokurtic distribution indicating that most effects are very small (Kim et al., 2017).

Thus, while there are some differences in the mutational parameters and rates of ID between species, the results of studies conducted in Drosophila can roughly be applied to other species. However, the estimation of the rate of ID is strongly influenced by the variation in *F* values among individuals, which is largely determined by the length of the genome. In particular, the small size of the Drosophila genome (140 Mb of euchromatin, with a genetic length of only about 3 Morgans, which effectively becomes 1.5 Morgans due to lack of recombination in males) is a peculiarity that affects the estimation of the rate of ID. It is expected that estimates of inbreeding coefficients will vary due to Mendelian sampling and linkage, with this variation being particularly significant for short genomes (Hill & Weir, 2011). For example, in the case of large genomes such as the human one, the expected standard deviation of the proportion of the autosomal genome shared between full siblings is approximately 0.04 (Hill & Weir, 2011; Visscher et al., 2006). However, this variation is much greater for short genomes (Hill & Weir, 2011). To illustrate this point, we conducted computer simulations and the results are presented in Supplemental File S4. Specifically, we calculated the standard deviation of molecular *F* values of offspring from full‐sibs using the estimator *F*_YAN_, for two genome sizes: a Drosophila‐like genome with two autosomal chromosomes of 0.5 Morgan each, and a mammal‐like genome with 20 chromosomes of one Morgan each. The standard deviation of molecular *F* values for the offspring of full sibs (expected *F* = 0.25) was 0.20 for the Drosophila‐like genome and 0.05 for the mammal‐like genome. Furthermore, the standard deviation of *F*_YAN_ values among unrelated individuals (expected *F* = 0) was about twice as high in the Drosophila scenario (around 0.04) compared to the mammal‐like genome (around 0.02). The larger variation in molecular *F* values among full siblings in Drosophila implies a greater power to estimate ID in this species, compared to larger genomes such as those of mammals. However, this effect is likely to be very small for unrelated non‐inbred individuals, for which the variation among *F* values is expected to be low for both types of genomes.

We used two different measures of inbreeding to estimate the inbreeding of individuals in our study. The first measure was the SNP‐by‐SNP estimator of inbreeding (*F*_YAN_), which has shown excellent performance in simulation studies and has been found to be highly accurate in estimating ID in various scenarios (Caballero et al., 2021, 2022; Nietlisbach et al., 2019; Yengo et al., 2017). In fact, *F*_YAN_ had the strongest correlations with both the phenotypes (Caballero et al., 2021; Yengo et al., 2018) and the homozygous mutation load (Alemu et al., 2021; Caballero et al., 2021), which has been suggested to be a proxy for fitness (Keller et al., 2011). We also considered estimates obtained from ROH fragments, which have been repeatedly shown to provide good estimates of ID (Caballero et al., 2021, 2022; Curik et al., 2014; Keller et al., 2011; Nietlisbach et al., 2019). In comparison, other measures of inbreeding from molecular data, such as those from Li and Horvitz (1953), VanRaden (2008), or the direct SNP homozygosity, are expected to produce more biased estimates of ID than *F*_YAN_ or *F*_ROH_ (Caballero et al., 2021, 2022).

Average values of the *F* estimates obtained with molecular data were generally consistent with expectations (Table 1). SNP‐by‐SNP‐based estimates provided deviations from Hardy–Weinberg proportions or correlations between alleles when the current allele frequencies were used instead of those from the base population, which are usually unknown (Wang, 2014). In random mated finite populations, the expected values are typically slightly negative due to the excess of heterozygotes, approximating −1/(2*N*) (Kimura & Crow, 1963). Larger positive values are expected due to the loss of heterozygosity as the proportion of full‐sib mating increases (Ghai, 1969). ROH‐based measures provided larger estimates of inbreeding, as expected, since they are supposed to capture more ancient inbreeding (Keller et al., 2011; Pryce et al., 2014). We observed a reduction in *F* estimates (and SEs) for an increase in the minimum length of ROH fragments, with *F*_ROH‐1_ values close to zero (0.0518 ± 0.0119) for individuals with *F*_PED_ = 0. Correlations between molecular *F* estimates were high for all estimators (Table 3), being generally above 0.8, in agreement with those obtained for several domestic populations (see a review of correlations in table 1 of Caballero et al., 2022). Despite the fact that only males were sequenced, and the phenotypic traits depended on both members of the pair (*P*) or even different pairs (*W*), we found that *F* estimates were highly negatively correlated with the phenotypes (pupae productivity and fitness), with correlations around −0.3 (Table 4).

Estimates of ID were obtained by regressing the phenotypic values on the molecular inbreeding coefficients. When considering a simple design of full‐sib mating progeny compared with non‐inbred progeny, pedigree‐based estimates of inbreeding depression are expected to be accurate. Therefore, we used *ID*_PED_ (obtained from all individuals with phenotypic values, either sequenced or not) as the standard ID measure against which we compared the molecular estimates. Because the two traits analyzed are complex ones (involving mating success, fecundity, and egg‐to‐pupae viability for *P*, plus egg‐to‐adult viability for *W*), they are expected to depend on both members of the pairs. As molecular measures of inbreeding were obtained only from males, the estimates of ID from these measures are expected to be 1/2 and 1/8 of those of reference, for *P* and *W*, respectively, in the case of non‐inbred samples (*F*_PED_ = 0; Supplemental File S2). For the inbred individuals, because these are the progeny of full sibs, the expected coancestry between the members of the evaluated pair would be *f* = 0.375, which implies that they share, on average, a 75% of their genome. Thus, the expected fractions of the ID to be estimated by markers was about 7/8 and 9/32 for *P* and *W* respectively (Supplemental File S2; Table 5). For productivity, the estimates of ID from *F*_YAN_ were rather close to the expectations for the set of non‐inbred individuals (*F*_PED_ = 0), whereas both *F*_YAN_ and *F*_ROH_ provided good estimates for the set of inbred individuals (*F*_PED_ = 0.25; Table 5). For fitness, however, the molecular estimates were generally larger than expectations probably because of the large error attached to the estimates.

Estimates of ID were higher for large ROH than for smaller ones (*F*_ROH‐1_ vs. *F*_ROH‐0.1_), which is consistent with empirical findings. For example, Pryce et al. (2014) reported ID for milk yield for Holstein and Jersey dairy cattle when considering long ROH, but no ID was detected using short ROH. Short ROH have originated from ancient common ancestors (i.e., ancient inbreeding), whose length has been reduced over generations due to recombination, which can lead to overestimations of the current genomic inbreeding. In addition, the effect of genetic purging, which removes deleterious mutations or reduces their frequency, could have mitigated the impact of ancient inbreeding on fitness. As a result, using short ROH may underestimate the extent of current ID. On the other hand, it is important to note that large ROH can also lead to overestimation of ID, as they only indicate recent inbreeding and may therefore underestimate the current genomic inbreeding. The estimates of ID for fitness (*W*) were found to be much more variable than those for productivity. This was expected, as the design of the study involved measuring inbreeding in a single male, while four couples were involved in the expression of the trait. Estimates from inbred individuals, in fact, were substantially larger than expected, and this could be explained by the above reason. For example, if the family from which the male was sequenced had lower inbreeding and higher fitness than the other three families, the reduced overall fitness observed would be associated with a small *F* value, which could lead to an overestimation of ID. The sequencing of all individuals in that group could have solved this uncertainty.

An interesting issue arising from Figures 2 and 3, is that, for a given *F* value, the fitness of inbred individuals (*F*_PED_ = 0.25) appears to be generally lower than that for non‐inbred ones (*F*_PED_ = 0). To investigate this, we compared individuals from the two sets with virtually identical *F* values estimated by *F*_ROH‐0.1_ (Table S7 in Supplemental File S3). Indeed, despite having the same *F* value, individuals with *F*_PED_ = 0.25 had lower average productivity and fitness than those with *F*_PED_ = 0. In addition, individuals with *F*_PED_ = 0.25 had in all comparisons a lower number of ROHs (32.8 on average) but longer (640.91 kb) than individuals with *F*_PED_ = 0 (44.8 fragments on average with an average length of 466.98 Mb), as expected. An explanation for this observation can be that ROH in the *F*_PED_ = 0 group, arising from more ancient inbreeding, had likely been subjected to more intense purging than longer ROH belonging to *F*_PED_ = 0.25 individuals, and arising from more recent inbreeding. This is in line with the observation in human data that long ROH are more enriched in deleterious mutations than short ones (Szpiech et al., 2013).

Simulation results obtained by Wang (2016) indicated that SNP‐by‐SNP based measures offer accurate ID estimates as long as the number of markers is high enough (around 10,000). We did not find large differences when reducing the number of SNPs in the mean of SNP‐by‐SNP‐based estimates of *F* and ID, although SEs increased considerably (Table S4, Supplemental File S3). However, ROH‐based estimates were the most affected, as expected. Previous studies comparing the use of SNPs obtained from complete sequences or from SNP chips indicated that a large number of SNPs is required to properly detect short IBD segments (Ferenčaković et al., 2013; Purfield et al., 2012; Zhang et al., 2015). In agreement with Ferenčaković et al. (2013), we observed an increase of *F*_ROH‐1_ for a decreasing number of SNPs up to 20,000, with a consequent decrease of ID. Whole genome sequencing provides information on rare alleles segregating at low frequencies, which can affect inbreeding estimates, particularly *F*_YAN_. Eynard et al. (2015) found significant differences on *F*_YAN_ when obtained from common SNPs (MAF ≥0.05) or from rare alleles (MAF ≥0.01) using whole genome sequence data from Holstein bulls. We did not find differences in *F*_YAN_ estimates by applying a MAF pruning, probably because the low sample size considered, although we did observe a slight reduction in the estimate of ID.

As a concluding remark, our results support the use of molecular markers in inferring inbreeding depression, even in extreme situations with a minimal sample size of unrelated individuals derived from a single generation, and when the inbreeding measures are only partially associated with the measured traits.

## CONFLICT OF INTEREST STATEMENT

We declare no conflict of interests.

## Supporting information


Data S1

Data S2

Data S3

Data S4

## Data Availability

Computer codes and scripts are available at Github address https://github.com/noeliaperezp/ID‐from‐molecular‐markers. Fastq files have been deposited in NCBI Short Read Archive under accession numbers SAMN34257684–SAMN34257717 (BioProject accession: PRJNA957565).
