# Supplementary material for: An empirical evaluation of the estimation of inbreeding depression from molecular markers under suboptimal conditions
Source: Evol Appl. 2023 Jun 28;16(7):1302–15. doi: 10.1111/eva.13568 (PMC10363801; doi:10.1111/eva.13568)
Supplement: Supplementary file 2 — Data S2 [file EVA-16-1302-s001.docx]

**SUPPLEMENTAL FILE 2**

**Approximated expected values of the inbreeding depression rate for the experimental design**

***Design for productivity: expected F_PED_ = 0***

Productivity is measured from pairs of individuals. Let assume that the productivity of the pair depends equally on the male and female parents, so that the productivity of the pair (*P*) is the average of the male and female productivities, i.e.

$P=\frac{P_{m}+P_{f}}{2}$.

The rate of inbreeding depression (*ID*), accounting for the mean productivity of the pair and their average inbreeding coefficients (*F*) is

$ID=\frac{cov\left( \frac{P_{m}+P_{f}}{2},\frac{F_{m}+F_{f}}{2} \right)}{var\left( \frac{F_{m}+F_{f}}{2} \right)}$ = $\frac{\frac{1}{4}\left[ cov\left( P_{m},F_{m} \right) + cov\left( P_{m},F_{f} \right) + cov\left( P_{f},F_{m} \right) + cov\left( P_{f},F_{f} \right) \right]}{\frac{1}{4}\left[ var\left( F_{m} \right) + var\left( F_{f} \right) \right]}$ .

In the case where the expected *F_PED_* = 0, i.e. the males and females from each pair are unrelated individuals, covariances between *P* of an individual and *F* of each pair are zero and, because there are not expected to be differences between sexes, $cov\left( P_{m},F_{m} \right)=cov\left( P_{f},F_{f} \right)=cov\left( P,F \right)$ and $var\left( F_{m} \right)=var\left( F_{f} \right)=var\left( F \right)$, so that

$ID=\frac{cov\left( P,F \right)}{var\left( F \right)}$ . (Eqn. 1)

In the productivity design, only the inbreeding of the male (*F_m_*) is considered. Therefore,

$ID=\frac{cov\left( \frac{P_{m}+P_{f}}{2},F_{m} \right)}{var\left( F_{m} \right)}$ = $\frac{\frac{1}{2}\left[ cov\left( P_{m},F_{m} \right) + cov\left( P_{f},F_{m} \right) \right]}{var\left( F_{m} \right)}$ ,

so that the rate of inbreeding depression is expected to be one half that when *F* is measured in both individuals of the pair,

$ID=\frac{1}{2}\frac{cov\left( P,F \right)}{var\left( F \right)}$ . (Eqn. 2)

***Design for productivity: expected F_PED_ = 0.25***

If now the pairs for which productivity is measured are full sibs which in turn are offspring from full-sib parents, the coefficient of coancestry between the individuals of the pair is expected to be *f* = 0.375 and their genetic relationship *r* = 0.75. Therefore, we may assume that $cov\left( P_{f},F_{m} \right)=\frac{3}{4}cov\left( P_{f},F_{f} \right)=\frac{3}{4}cov\left( P,F \right)$. Then,

$ID=\frac{cov\left( \frac{P_{m}+P_{f}}{2}, F_{m} \right)}{var\left( F_{m} \right)}$ = $\frac{\frac{1}{2}\left[ cov\left( P_{m},F_{m} \right) + cov\left( P_{f}, F_{m} \right) \right]}{var\left( F_{m} \right)}$ = $\frac{\frac{1}{2}\left[ cov\left( P,F \right) + \frac{3}{4}cov\left( P,F \right) \right]}{var\left( F_{m} \right)}$ ,

and,

$ID=\frac{7}{8}\frac{cov\left( P,F \right)}{var\left( F \right)}$ . (Eqn. 3)

***Design for competitive fitness: expected F_PED_ = 0***

In the competitive fitness design, fitness is measured in the female progeny from four pairs of individuals. If we assume that the fitness measure depends equally on all four pairs of unrelated parents but we only measure inbreeding in one of the males (individual *i* = 1; i.e., *F*_1_),

$ID=\frac{cov\left( \frac{1}{8}\sum_{i} W_{i},F_{1} \right)}{var\left( F_{1} \right)}$ = $\frac{\frac{1}{8}\left[ cov\left( W_{1},F_{1} \right) + \sum_{i\neq1} cov\left( W_{i},F_{1} \right) \right]}{var\left( F_{1} \right)}$ ,

and because all individuals in the four pairs are unrelated,

$ID=\frac{1}{8}\frac{cov\left( W,F \right)}{var\left( F \right)}$ . (Eqn. 4)

***Design for competitive fitness: expected F_PED_ = 0.25***

Finally, in the competitive fitness (*W*) design, assuming all pairs are full-sib offspring from full sibs, being *i* = 1 the male which inbreeding coefficient is estimated, and *i* = 2 the corresponding female pair,

$ID=\frac{cov\left( \frac{1}{8}\sum_{i} W_{i},F_{1} \right)}{var\left( F_{1} \right)}$ = $\frac{\frac{1}{8}\left[ cov\left( W_{1},F_{1} \right) + cov\left( W_{2},F_{1} \right) + \sum_{i>2} cov\left( W_{i},F_{1} \right) \right]}{var\left( F_{1} \right)}$ = $\frac{\frac{1}{8}\left[ cov\left( W,F \right) + \frac{3}{4}cov\left( W,F \right) \right]}{var\left( F_{1} \right)}$ ,

so that,

$ID=\frac{7}{32}\frac{cov\left( W,F \right)}{var\left( F \right)}$ . (Eqn. 5)

Now, in the actual breeding design it was not possible to include unrelated pairs of full sibs, and some of the full-sib pairs were cousins of another full-sib pair. If we assume that the pair formed by the male *i* = 1 and the female *i* = 2 are cousins of the pair formed by the male *i* = 3 and the female *i* = 4, the coancestry coefficient between individual *i* = 1 and individuals *i* = 3 or 4 is *f* = 0.25, so their genetic relationship coefficient is *r* = 0.5. Thus, $cov\left( W_{3},F_{1} \right)=cov\left( W_{4},F_{1} \right)=\frac{1}{2}cov\left( W_{1},F_{1} \right)=\frac{1}{2}cov\left( W,F \right)$. Then,

$ID=\frac{cov\left( \frac{1}{8}\sum_{i} W_{i},F_{1} \right)}{var\left( F_{1} \right)}$ = $\frac{\frac{1}{8}\left[ cov\left( W_{1},F_{1} \right) + cov\left( W_{2},F_{1} \right) + cov\left( W_{3},F_{1} \right) + cov\left( W_{4},F_{1} \right) + \sum_{i>4} cov\left( W_{i},F_{1} \right) \right]}{var\left( F_{1} \right)}$ = $\frac{\frac{1}{8}\left[ cov\left( W,F \right) + \frac{3}{4}cov\left( W,F \right) + \frac{1}{2}cov\left( W,F \right) + \frac{1}{2}cov\left( W,F \right) \right]}{var\left( F_{1} \right)}$ ,

and

$ID=\frac{11}{32}\frac{cov\left( W,F \right)}{var\left( F \right)}$ . (Eqn. 6)

Because about half the full-sib pairs were cousins of another full-sib pairs in the final breeding design, the expected ID is the average between equations (5) and (6), i.e.,

$ID=\frac{9}{32}\frac{cov\left( W,F \right)}{var\left( F \right)}$ . (Eqn. 7)
