# Supplementary material for: An empirical evaluation of the estimation of inbreeding depression from molecular markers under suboptimal conditions
Source: Evol Appl. 2023 Jun 28;16(7):1302–15. doi: 10.1111/eva.13568 (PMC10363801; doi:10.1111/eva.13568)
Supplement: Supplementary file 3 — Data S3 [file EVA-16-1302-s004.docx]

**SUPPLEMENTAL FILE 3**

The following results are shown in this file:

- **Tables S1-S3:** Probability values for inbreeding depression (ID) estimates and for Pearson correlations between *F* estimates and phenotypic values, as well as between *F* estimates with each other for individuals with *F_PED_* = 0 and *F_PED_* = 0.25 separately. Significant probabilities are shown in bold face for all tables.
- **Tables S4 and S5:** Following the same methodology as that detailed in the main text, further inbreeding analyses were performed based on different numbers of SNPs used for the estimations. Average *F* and ID (for individuals with *F_PED_* = 0 and *F_PED_* = 0.25 separately) were estimated from a number of SNPs (5,000, 10,000, 20,000, 50,000, 100,000 and 500,000) taken at random with the command *--extract* from PLINK (version 1.9) from a list of random SNPs previously obtained with the Linux *shuf –n* command using the *map* file as input. In addition, a minor allele frequency (MAF) of 0.05 was applied to estimate molecular *F* values and ID estimates.
- **Table S6:** Estimates of *F* and ID were obtained after modifying some PLINK parameters for ROH detection.
- **Table S7:** Comparison between individuals of the two sets (*F_PED_* = 0 and *F_PED_* = 0.25) showing the same estimate of inbreeding.

**Table S1.** Probability values for Pearson correlations between different molecular estimates of *F* for the two sets of individuals (non-inbred, *F_PED_* = 0 over the diagonal; and inbred, *F_PED_* = 0.25, below the diagonal).

|  | ***F_YAN_*** | ***F_ROH_*_-0.1_** | ***F_ROH_*_-1_** |
| --- | --- | --- | --- |
| ***F_YAN_*** | - | **6.8 × 10^–6^** | **2.6 × 10^–4^** |
| ***F_ROH_*_-0.1_** | **3.3 × 10^–6^** | - | **6.0 × 10^–7^** |
| ***F_ROH_*_-1_** | **3.2 × 10^–6^** | **1.9 × 10^–10^** | - |

**Table S2.** Probability values for Pearson correlations between the phenotypes and the molecular estimates of *F*.

|  | ***F_YAN_*** | ***F_ROH_*_-0.1_** | ***F_ROH_*_-1_** |
| --- | --- | --- | --- |
| ***F_PED_* = 0** |  |  |  |
| **Log(*P*)** | 0.113 | 0.404 | 0.499 |
| **Log(*W*)** | 0.555 | 0.900 | 0.666 |
| ***F_PED_* = 0.25** |  |  |  |
| **Log(*P*)** | 0.199 | 0.175 | 0.246 |
| **Log(*W*)** | 0.082 | 0.090 | 0.118 |

**Table S3.** Probability values for inbreeding depression estimates obtained with a parametric one-tail test, a randomization test, and a bootstrap test (see main text for details).

| **Trait** | ***F_PED_*** | **Test** | ***ID_PED_*** | ***ID_YAN_*** | ***ID_ROH-_*_0.1_** | ***ID_ROH-_*_1_** |
| --- | --- | --- | --- | --- | --- | --- |
| log(*P*) | 0 | Parametric | **6.3 × 10^–9^** | 0.056 | 0.202 | 0.249 |
|  |  | Randomization | **10^–4^** | 0.063 | 0.199 | 0.250 |
|  |  | Bootstrap | **0** | **0.030** | 0.170 | 0.250 |
|  | 0.25 | Parametric |  | 0.099 | 0.088 | 0.123 |
|  |  | Randomization |  | 0.104 | 0.091 | 0.134 |
|  |  | Bootstrap |  | 0.080 | 0.110 | 0.150 |
|  | Both | Parametric |  | **0.002** | **0.008** | **0.008** |
|  |  | Randomization |  | **0.004** | **0.010** | **0.011** |
|  |  | Bootstrap |  | **0.012** | **0.030** | **0.041** |
| log(*W*) | 0 | Parametric | **0.007** | 0.278 | 0.450 | 0.333 |
|  |  | Randomization | **0.007** | 0.271 | 0.449 | 0.333 |
|  |  | Bootstrap | **0.003** | 0.240 | 0.450 | 0.310 |
|  | 0.25 | Parametric |  | **0.041** | **0.045** | 0.059 |
|  |  | Randomization |  | **0.042** | **0.043** | 0.056 |
|  |  | Bootstrap |  | 0.060 | **0.050** | 0.090 |
|  | Both | Parametric |  | **0.003** | **0.011** | **0.006** |
|  |  | Randomization |  | **0.003** | **0.009** | **0.005** |
|  |  | Bootstrap |  | **0.005** | **0.008** | **0.004** |

**Table S4.** Mean inbreeding coefficient (*F* ± standard error) obtained from different molecular measures. Default values correspond to those presented in the main text.

|  | ***F_YAN_*** | ***F_ROH_*_–0.1_** | ***F_ROH_*_–1_** |
| --- | --- | --- | --- |
| ***F_PED_* = 0** |  |  |  |
| ***Default*** | −0.0520 ± 0.0154 | 0.1477 ± 0.0181 | 0.0518 ± 0.0119 |
| **MAF = 0.05** | −0.0562 ± 0.0181 | 0.1457 ± 0.0175 | 0.0529 ± 0.0119 |
| **500,000 SNPs^a^** | −0.0520 ± 0.0154 | 0.1490 ± 0.0180 | 0.0702 ± 0.0138 |
| **100,000 SNPs^b^** | −0.0520 ± 0.0155 | 0.1484 ± 0.0190 | 0.1039 ± 0.0181 |
| **50,000 SNPs^c^** | −0.0521 ± 0.0155 | 0.1424 ± 0.0194 | 0.1108 ± 0.0191 |
| **20,000 SNPs^c^** | −0.0519 ± 0.0154 | 0.1270 ± 0.0198 | 0.1128 ± 0.0197 |
| **10,000 SNPs^c^** | −0.0521 ± 0.0155 | 0.0866 ± 0.0188 | 0.0852 ± 0.0187 |
| **5,000 SNPs^c^** | −0.0512 ± 0.0156 | 0.0334 ± 0.0100 | 0.0334 ± 0.0100 |
| ***F_PED_* = 0.25** |  |  |  |
| ***Default*** | 0.0346 ± 0.0245 | 0.2143 ± 0.0256 | 0.1181 ± 0.0199 |
| **MAF = 0.05** | 0.0469 ± 0.0288 | 0.2109 ± 0.0251 | 0.1200 ± 0.0201 |
| **500,000 SNPs^a^** | 0.0346 ± 0.0245 | 0.2149 ± 0.0253 | 0.1415 ± 0.0231 |
| **100,000 SNPs^b^** | 0.0347 ± 0.0245 | 0.2119 ± 0.0265 | 0.1728 ± 0.0271 |
| **50,000 SNPs^c^** | 0.0342 ± 0.0245 | 0.2071 ± 0.0277 | 0.1787 ± 0.0283 |
| **20,000 SNPs^c^** | 0.0349 ± 0.0247 | 0.1941 ± 0.0295 | 0.1818 ± 0.0296 |
| **10,000 SNPs^c^** | 0.0351 ± 0.0248 | 0.1542 ± 0.0255 | 0.1531 ± 0.0255 |
| **5,000 SNPs^c^** | 0.0344 ± 0.0249 | 0.1131 ± 0.0215 | 0.1131 ± 0.0215 |

^a^ 5 replicates; ^b^ 10 replicates; ^c^ 20 replicates. Standard errors were calculated for each replicate and then averaged over replicates.

**Table S5.** Inbreeding depression rate (ID) obtained from different molecular measures of inbreeding for two traits, pupae productivity (log(*P*)) and fitness (log(*W*)). Default values correspond to those presented in the main text.

|  | ***F_PED_ =* 0** | | |  | ***F_PED_ =* 0.25** | | |
| --- | --- | --- | --- | --- | --- | --- | --- |
| **Log(*P*)** | Expected = –1.64 | | |  | Expected = –2.86 | | |
|  | ***ID_YAN_*** | ***ID_ROH_*_–0.1_** | ***ID_ROH_*_–1_** |  | ***ID_YAN_*** | ***ID_ROH_*_–0.1_** | ***ID_ROH_*_–1_** |
| **Default** | –1.884 | –0.871 | –1.077 |  | –2.419 | –2.440 | –2.705 |
| **MAF = 0.05** | –1.710 | –0.855 | –1.109 |  | –2.088 | –2.506 | –2.697 |
| **500,000 SNPs^a^** | –1.877 ± 0.007 | –0.819 ± 0.013 | –1.205 ± 0.103 |  | –2.414 ± 0.012 | –2.510 ± 0.003 | –2.586 ± 0.094 |
| **100,000 SNPs^b^** | –1.883 ± 0.018 | –0.745 ± 0.015 | –0.841 ± 0.022 |  | –2.421 ± 0.019 | –2.505 ± 0.031 | –2.361 ± 0.052 |
| **50,000 SNPs^c^** | –1.875 ± 0.017 | –0.608 ± 0.024 | –0.682 ± 0.026 |  | –2.399 ± 0.019 | –2.471 ± 0.028 | –2.363 ± 0.030 |
| **20,000 SNPs^c^** | –1.865 ± 0.029 | –0.733 ± 0.035 | –0.803 ± 0.038 |  | – 2.396 ± 0.022 | –2.364 ± 0.032 | –2.359 ± 0.030 |
| **10,000 SNPs^c^** | –1.852 ± 0.029 | –1.608 ± 0.083 | –1.607 ± 0.084 |  | –2.344 ± 0.033 | –2.082 ± 0.094 | –2.075 ± 0.094 |
| **5,000 SNPs^c^** | –1.835 ± 0.051 | –3.447 ± 0.114 | –3.447 ± 0.114 |  | –2.396 ± 0.049 | –1.520 ± 0.063 | –1.520 ± 0.063 |
| **Log(*W*)** | Expected = –0.47 | | |  | Expected = –1.05 | | |
|  | ***ID_YAN_*** | ***ID_ROH_*_–0.1_** | ***ID_ROH_*_–1_** |  | ***ID_YAN_*** | ***ID_ROH_*_–0.1_** | ***ID_ROH_*_–1_** |
| **Default** | –1.946 | –0.355 | –1.847 |  | –5.504 | –5.157 | –6.154 |
| **MAF = 0.05** | –2.149 | –0.034 | –1.639 |  | –4.875 | –5.201 | –6.071 |
| **500,000 SNPs^a^** | –1.938 ± 0.016 | –0.015 ± 0.059 | –1.074 ± 0.196 |  | –5.500 ± 0.012 | –5.210 ± 0.018 | –5.517 ± 0.216 |
| **100,000 SNPs^b^** | –1.946 ± 0.036 | +0.078 ± 0.067 | –0.214 ± 0.094 |  | –5.541 ± 0.030 | –4.946 ± 0.073 | –4.700 ± 0.072 |
| **50,000 SNPs^c^** | –2.012 ± 0.044 | +0.325 ± 0.055 | +0.045 ± 0.075 |  | –5.514 ± 0.022 | –4.753 ± 0.036 | –4.539 ± 0.030 |
| **20,000 SNPs^c^** | –2.019 ± 0.046 | +0.031 ± 0.066 | –0.139 ± 0.076 |  | –5.484 ± 0.056 | –4.531 ± 0.042 | –4.401 ± 0.038 |
| **10,000 SNPs^c^** | –1.976 ± 0.088 | –1.792 ± 0.171 | –1.793 ± 0.181 |  | –5.338 ± 0.065 | –4.726 ± 0.086 | –4.748 ± 0.089 |
| **5,000 SNPs^c^** | –2.087 ± 0.110 | –2.257 ± 0.547 | –2.257 ± 0.547 |  | –5.522 ± 0.119 | –4.104 ± 0.107 | –4.104 ± 0.107 |

^a^ 5 replicates; ^b^ 10 replicates; ^c^ 20 replicates.

**Table S6.** Mean inbreeding coefficient (*F* ± standard error) and inbreeding depression rate (ID) obtained from ROH with a minimum length of 0.1 Mb and different PLINK parameters. Default (def.) values correspond to those presented in the main text.

|  | ***F* = 0** | | |  | ***F* = 0.25** | | |
| --- | --- | --- | --- | --- | --- | --- | --- |
|  | ***F_ROH_*_–0.1_** | ***ID_ROH_*_–0.1_ (*P*)** | ***ID_ROH_*_–0.1_ (*W*)** |  | ***F_ROH_*_–0.1_** | ***ID_ROH_*_–0.1_ (*P*)** | ***ID_ROH–0.1_* (*W*)** |
| **Expected** |  | **–1.640** | **–0.470** |  |  | **–2.860** | **–1.050** |
| **Default parameters** | **0.1477 ± 0.0181** | **–0.871** | **–0.355** |  | **0.2143 ± 0.0256** | **–2.440** | **–5.157** |
| *--homozyg-density* ^(def. 50)^ |  |  |  |  |  |  |  |
| 70 kb/SNP | 0.1477 ± 0.0181 | –0.871 | –0.355 |  | 0.2143 ± 0.0256 | –2.440 | –5.157 |
| 5 kb/SNP | 0.1468 ± 0.0178 | –0.858 | –0.200 |  | 0.2127 ± 0.0253 | –2.482 | –5.191 |
| 1 kb/SNP | 0.1389 ± 0.0159 | –0.997 | +0.213 |  | 0.2024 ± 0.0239 | –2.845 | –5.685 |
| *--homozyg-gap* ^(def. 1000)^ |  |  |  |  |  |  |  |
| 500 kb | 0.1477 ± 0.0181 | –0.871 | –0.355 |  | 0.2143 ± 0.0256 | –2.440 | –5.157 |
| 50 kb | 0.1465 ± 0.0177 | –0.878 | –0.257 |  | 0.2128 ± 0.0254 | –2.485 | –5.149 |
| 10 kb | 0.1378 ± 0.0162 | –0.980 | +0.269 |  | 0.2036 ± 0.0240 | –2.677 | –5.502 |
| *--homozyg-window-snp* ^(def.50)^ |  |  |  |  |  |  |  |
| 100 SNPs | 0.1433 ± 0.0172 | –0.902 | –0.016 |  | 0.2089 ± 0.0249 | –2.451 | –5.235 |
| 60 SNPs | 0.1476 ± 0.0181 | –0.869 | –0.339 |  | 0.2128 ± 0.0255 | –2.488 | –5.179 |
| 10 SNPs | 0.1570 ± 0.0192 | –0.623 | +0.061 |  | 0.2244 ± 0.0265 | –2.446 | –4.909 |
| *--homozyg-snp* ^(def. 100)^ |  |  |  |  |  |  |  |
| 60 SNPs | 0.1497 ± 0.0183 | –0.819 | –0.246 |  | 0.2162 ± 0.0257 | –2.413 | –5.059 |
| 30 SNPs | 0.1513 ± 0.0182 | –0.741 | –0.088 |  | 0.2169 ± 0.0258 | –2.418 | –5.030 |

**Table S7.** Comparison between individuals belonging to the set with *F_PED_* = 0 and to the set with *F_PED_* = 0.25 with almost identical estimated inbreeding values. The columns show, for each individual (ind; see codes in Supplemental File S1), the estimate of inbreeding based on ROH fragments larger than 0.1 Mb, the number of ROH, the average ROH length, the total length of ROH regions in the genome, and the estimated productivity and fitness values. Average values and standard errors for the five sets are shown at the end of the table.

|  | ***F_ROH-0.1_*** | **Number of ROH** | **ROH lenght** | **Total lenght** | **log(*P*)** | **log(*W*)** |
| --- | --- | --- | --- | --- | --- | --- |
| ***Comparison 1*** |  |  |  |  |  |  |
| *F_PED_* = 0 (ind. B40) | 0.098 | 25 | 468.34 | 11709 | 4.466 | 0.440 |
| *F_PED_* = 0.25 (ind. F1B) | 0.099 | 19 | 617.69 | 11736 | 4.382 | 1.455 |
| ***Comparison 2*** |  |  |  |  |  |  |
| *F_PED_* = 0 (ind. B20) | 0.109 | 34 | 382.87 | 13018 | 4.762 | 0.397 |
| *F_PED_* = 0.25 (ind. F9A) | 0.118 | 30 | 466.53 | 13996 | 2.890 | -0.560 |
| ***Comparison 3*** |  |  |  |  |  |  |
| *F_PED_* = 0 (ind. B78) | 0.177 | 55 | 382.49 | 21037 | 4.673 | 0.909 |
| *F_PED_* = 0.25 (ind. F20B) | 0.178 | 37 | 572.3 | 21175 | 4.511 | -0.780 |
| ***Comparison 4*** |  |  |  |  |  |  |
| *F_PED_* = 0 (ind. B5) | 0.245 | 52 | 561.74 | 29210 | 3.970 | -1.112 |
| *F_PED_* = 0.25 (ind. F40A) | 0.244 | 40 | 725.3 | 29012 | 3.611 | -0.361 |
| ***Comparison 5*** |  |  |  |  |  |  |
| *F_PED_* = 0 (ind. B64) | 0.263 | 58 | 539.45 | 31288 | 4.564 | 0.909 |
| *F_PED_* = 0.25 (ind. F69B) | 0.263 | 38 | 822.74 | 31264 | 3.989 | -0.274 |
| **AVERAGES ± SE** |  |  |  |  |  |  |
| ***F_PED_* = 0** | **0.178**  ± 0.034 | **44.8**  ± 6.476 | **466.98**  ± 37.71 | **21252**  ± 4018 | **4.487**  ± 0.139 | **0.309**  ± 0.372 |
| ***F_PED_* = 0.25** | **0.180**  ± 0.033 | **32.8**  ± 3.839 | **640.91**  ± 61.58 | **21437**  ± 3895 | **3.877**  ± 0.293 | **-0.104**  ± 0.400 |
