# Supplementary material for: An empirical evaluation of the estimation of inbreeding depression from molecular markers under suboptimal conditions
Source: Evol Appl. 2023 Jun 28;16(7):1302–15. doi: 10.1111/eva.13568 (PMC10363801; doi:10.1111/eva.13568)
Supplement: Supplementary file 4 — Data S4 [file EVA-16-1302-s003.docx]

**SUPPLEMENTAL FILE 4**

**Computer simulations**

To investigate the magnitude of the variance of molecular estimates of inbreeding in different scenarios, we carried out computer simulations using the software SLiM (Haller and Messer, 2019) and an in-house C programme. SLiM simulates an individually-based forward Wright-Fisher model of reproduction. We simulated populations of constant size (*N* = 1,000 individuals) with random mating for 10,000 discrete generations, assuming no selection. Two scenarios were considered: a Drosophila-like scenario with two autosomal chromosomes of 50 Mb each and a mammal-like scenario with 20 autosomal chromosomes of 100 Mb each. A rate of recombination between nucleotides of 10^–8^ was assumed, which implies a chromosome length of 0.5 Morgan for the Drosophila-like scenario and one Morgan for the mammal-like scenario. The rate of mutation per nucleotide was set to 10^–8^ - 10^–9^ to obtain at least 50,000 SNPs for analysis. At the last generation, we used the C programme to sample 40 individuals from the previously simulated population and carry out 5 generations of full-sib mating. Each pair of individuals produced a pair of full sibs, which were then mated with each other to produce the next generation, accounting for recombination and drift. Mutation was not considered during these five generations. Using the estimator *F_YAN_* and assuming the current allele frequencies of SNPs, the inbreeding coefficient of individuals was estimated in all generations. The mean and standard deviation of *F* values was obtained for the 40 simulated individuals, and the process was repeated 1000 times, averaging the results over replicates.

The results for the Drosophila-like and mammal-like scenarios are shown in Figures S1 and S2, respectively. Graph (a) displays the mean estimate of the inbreeding coefficient for each consecutive generation of full-sib mating, with the average standard deviation of individual *F* values indicated by the bar above and below the mean. Graph (b) illustrates the distribution of individual *F* values at generation 2, where the expected inbreeding coefficient is 0.25. In this case, the average standard deviation of *F* values is 0.20 for the Drosophila-like scenario and 0.05 for the mammal-like scenario. At generation 0, where individuals are non-inbred and unrelated, the corresponding standard deviations are 0.08 and 0.04, respectively.


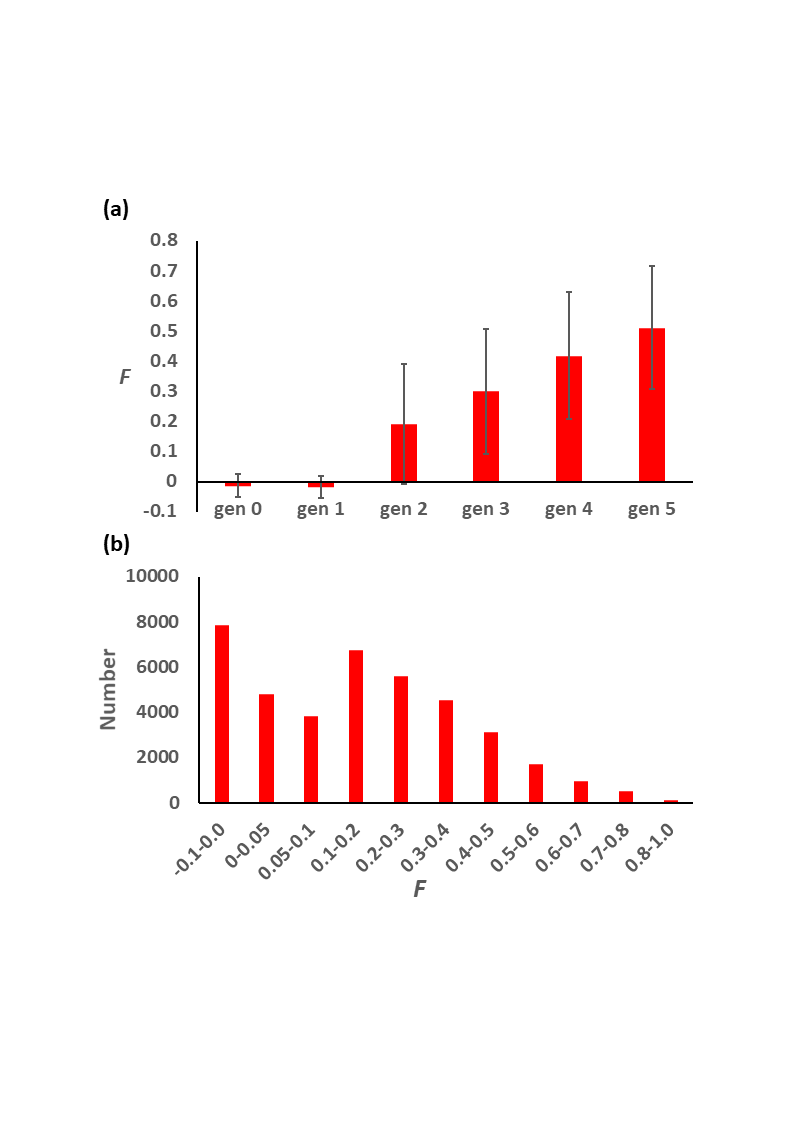


**Figure S1.** Simulations corresponding to a Drosophila-like scenario. (a) Mean inbreeding coefficient (*F*) obtained from SNP data using the estimator *F_YAN_* for each of five consecutive generations of full-sib mating. The bars above and below the mean indicate one standard deviation of the estimated *F* values among individuals. (b) Average distribution of estimated *F* values of individuals at generation 2, for which the expected inbreeding coefficient is 0.25.


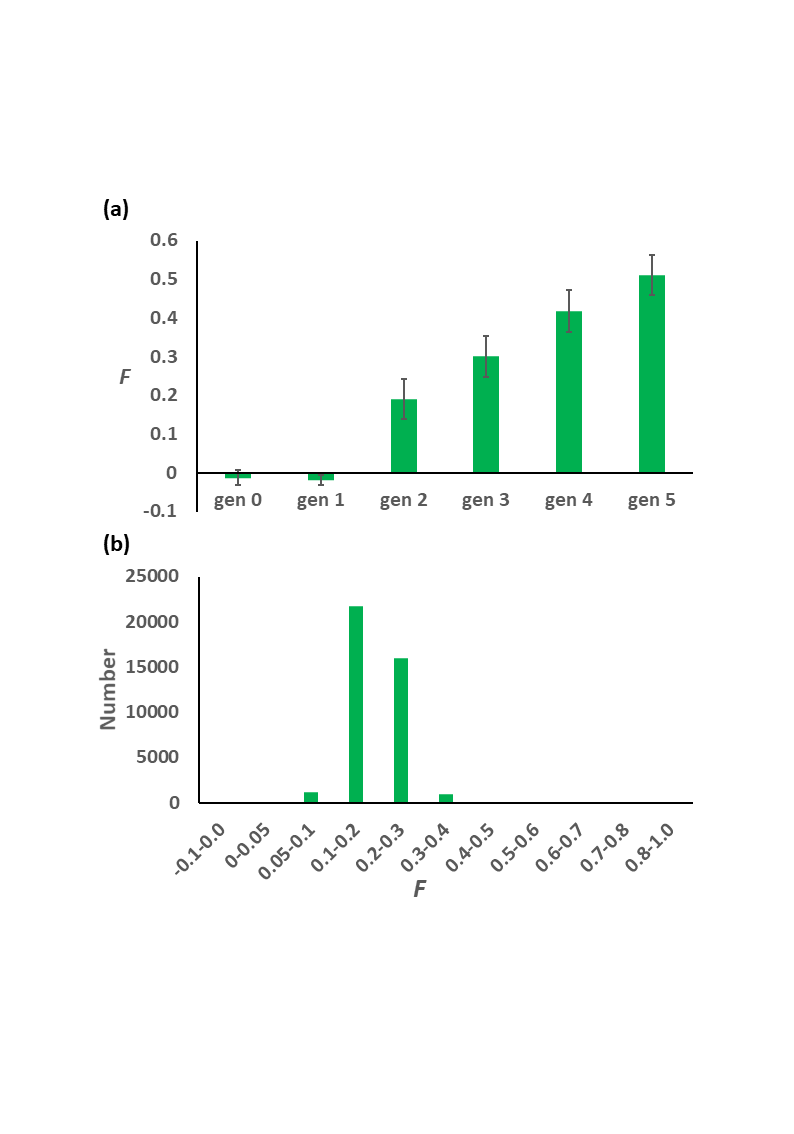


**Figure S2.** Simulations corresponding to a mammal-like scenario. (a) Mean inbreeding coefficient (*F*) obtained from SNP data using the estimator *F_YAN_* for each of five consecutive generations of full-sib mating. The bars above and below the mean indicate one standard deviation of the estimated *F* values among individuals. (b) Average distribution of estimated *F* values of individuals at generation 2, for which the expected inbreeding coefficient is 0.25.
